# Adipogenesis, Osteogenesis, and Chondrogenesis of Human Mesenchymal Stem/Stromal Cells: A Comparative Transcriptome Approach

**DOI:** 10.3389/fcell.2020.00561

**Published:** 2020-07-08

**Authors:** Anny W. Robert, Bruna H. Marcon, Bruno Dallagiovanna, Patrícia Shigunov

**Affiliations:** Instituto Carlos Chagas – Fiocruz Paraná, Curitiba, Brazil

**Keywords:** transcriptome, adipogenesis, osteogenesis, chondrogenesis, mesenchymal stem/stromal cell, gene expression profile, cell differentiation

## Abstract

Adipogenesis, osteogenesis and chondrogenesis of human mesenchymal stem/stromal cells (MSC) are complex and highly regulated processes. Over the years, several studies have focused on understanding the mechanisms involved in the MSC commitment to the osteogenic, adipogenic and/or chondrogenic phenotypes. High-throughput methodologies have been used to investigate the gene expression profile during differentiation. Association of data analysis of mRNAs, microRNAs, circular RNAs and long non-coding RNAs, obtained at different time points over these processes, are important to depict the complexity of differentiation. This review will discuss the results that were highlighted in transcriptome analyses of MSC undergoing adipogenic, osteogenic and chondrogenic differentiation. The focus is to shed light on key molecules, main signaling pathways and biological processes related to different time points of adipogenesis, osteogenesis and chondrogenesis.

## Introduction

Stem cells are undifferentiated cells that are capable of self-renew and, under appropriate stimuli, differentiate into specific cell lineages (Weissman, 2015). Adult stem cells can be found in all tissues from an adult organism. Because they don’t show the biological adverse effects of embryonic stem cells, can be used in autologous transplants and has fewer ethical issues, they have been the focus of basic and clinical research aiming their use in cell-based therapies (Dulak et al., 2015; Visvader and Clevers, 2016).

Mesenchymal stem/stromal cells (MSC) define a specific population of adult stem cells with specific characteristics that make them of high interest for clinical applications. MSC have been used in more than a thousand clinical trials for a wide range of diseases and clinical conditions (ClinicalTrials.gov). MSC have been described as fibroblastic precursors that can be isolated from bone marrow and that were able to differentiate into mesodermal-derived cells (Friedenstein et al., 1966). MSC are mesoderm-derived undifferentiated cells which also show the ability to self-renew and differentiate into a defined set of cell types. When stimulated both *in vivo* or *in vitro* they can differentiate into several mesodermal-derived lineages, in particular chondrogenic, osteogenic and adipogenic cells. Several reports indicate that these cells can also differentiate into non-mesodermal lineages like hepatocytes, neurons and pancreatic cells (Aurich et al., 2009; Marappagounder et al., 2013; Ghorbani et al., 2018).

Besides its fibroblast-like morphology and the capacity to differentiate in adipocytes, osteocytes and chondrocytes, MSC are defined based on a set of specific surface markers. In 2006, the International Society for Cellular Therapy (ISCT), propose the following phenotypic characteristics for defining MSC: more than 95% of the cells should express the surface proteins CD105, CD73 and CD90, and less than 2% of cells should be positive for the surface markers CD45, CD34, CD14 or CD11b, CD19 or CD79α, and HLA-DR. The set of negative markers avoid contamination with cells from hematopoietic lineage (Dominici et al., 2006). Considering the different sources of MSC, in 2013, the ISCT stated that to characterize mesenchymal/stromal cells isolated from adipose tissue (Bourin et al., 2013). In addition to the positive markers already described (Dominici et al., 2006), others such as CD13, CD29, CD44 (>80% positive cells) can also be included; in relation to the negative ones, CD31 and CD235a could be used. Other markers were also described, but with higher variation in its expression depending on culture conditions and passages (Bourin et al., 2013).

Furthermore, research groups had studied other markers, as STRO-1, CD146, CD271, SSEA-4, CD49f among others, which can be used, e.g., to differentiate populations of stem cells with different potentials (reviewed by Lv et al., 2014; Samsonraj et al., 2017). Despite the advances, controversies still remain regarding the ideal marker or set of markers, since many of them are expressed by other cell types and there may be changes in expression depending on the source or culture method of the MSC. Concerning these differences, the characterization of 246 surface markers in bone marrow and umbilical cord blood-derived MSC showed that both of them highly expressed 18 markers, including the classical ones (CD90, CD105, and CD73) as well as alpha-smooth muscle antigen (SMA), CD13, CD140b, CD276, CD29, CD44, CD59, CD81, CD98, HLA-ABC, and others (Amati et al., 2018). On the other hand, looking for markers that were differentially expressed, it was found that CD143 (an angiotensin-converting enzyme) was highly expressed in bone marrow and adipose tissue-derived MSC in comparison with umbilical cord blood and umbilical cord-derived MSC, suggesting that this marker could differentiate MSC from adult tissues and those derived from perinatal tissues (Amati et al., 2018). In relation to the influence of passage number, analysis of adipose tissue-derived MSC at passages #1 to #8 showed that they changed its immunophenotypic profile based on passage number, although some of the markers presented a variable expression independently from time (Peng et al., 2020).

Mesenchymal stem/stromal cells exist in various tissues being the bone marrow, adipose tissue and umbilical cord blood the preferred source of cells in both basic and clinical research. Their multilineage differentiation potential and their capacity to proliferate *in vitro*, make these cells of great value for tissue engineering (Pittenger et al., 1999; Rebelatto et al., 2008). Though increasing evidence points to a paracrine and an immunomodulatory effect as also responsible for the positive results observed in cell therapies (Gallina et al., 2015; Kuo et al., 2016; Caplan, 2017), their potential to proliferate, differentiate and repopulate the target organ is still a first choice to reconstruct the damaged tissue (Shao et al., 2015; Bacakova et al., 2018; Mazini et al., 2019; Gomez-Salazar et al., 2020).

Cell therapies using adult MSC are slowly being approved for a wide range of diseases, involving different protocols for isolation and commitment to differentiation in specific cell types. Understanding the regulatory pathways and factors involved in the commitment to a specific cell type and understanding the mechanisms that regulate proliferation and differentiation is essential for improvement and successful therapies (Gomez-Salazar et al., 2020).

A cascade of events occurs in the MSC during the differentiation process, generating phenotypic and metabolic transformations. The reduction of the expression of stemness genes and the activation of genes related to the function of a mature phenotype are the first steps of a cascade that will lead to a morphological alteration of the cell. Different studies demonstrated that factors and pathways that stimulate adipogenesis inhibit osteogenesis. Conversely, adipogenic induction inhibits osteogenesis (Beresford et al., 1992; James et al., 2010). The balance between adipogenesis and osteogenesis is important to keep homeostasis in the organism.

In healthy bones, there is a constant process of bone resorption mainly promoted by osteoclasts and the generation of new tissue by osteoblasts. While osteoclasts are derived from the hematopoietic lineage, being formed by the fusion of progenitors from the monocyte/macrophage family (Teitelbaum, 2000), osteoblasts are derived from bone marrow- derived MSC, which may also differentiate into adipogenic lineage (Owen, 1988; Caplan, 1991; Kokabu et al., 2016). An imbalance leading the bone marrow MSC toward a higher rate of adipogenic differentiation to the detriment of osteogenesis is associated with loss of bone mass and diseases, as osteoporosis (Chen et al., 2016; Kokabu et al., 2016).

Moreover, the multilineage potential and the ability to secrete immunomodulatory factors and other signaling molecules made MSC an important source for use in regenerative medicine. Innumerous approaches used MSC as a therapeutic alternative for diverse health problems, which includes, e.g., treatment of obesity (reviewed by Matsushita and Dzau, 2017; Saleh et al., 2018) and for bone repair (reviewed by Shao et al., 2015; Jin and Lee, 2018; Iaquinta et al., 2019), but the mechanism of action of MSC in the body has not yet been fully elucidated, just as many challenges remain in an attempt to improve the proposed therapeutic strategies.

In an attempt to improve our understanding of MSC and its differentiation processes, which could contribute to the development of new therapeutic approaches, several studies have focused on understanding the mechanisms involved in the fate decision of MSC toward adipogenic, osteogenic or chondrogenic differentiation. But despite the advances, these differentiation processes are not completely understood.

The cellular transcriptome can be defined as the total population of RNA molecules in the cell at a particular moment. Measuring the abundance of these transcripts allows us to define which genes are being expressed, and at what level, under a defined condition. The fate of a stem cell is directed by the gene expression profile at a particular moment and by the interactions among these transcripts and/or transcripts’ products (Billing et al., 2016; Hasin et al., 2017; Melamed, 2020). Our understanding of the dynamics of the cell transcriptome was only possible with the emergence of high throughput techniques to characterize the gene expression profile of a cell. At the onset of the century, transcriptomic studies used hybridization-based techniques, such as gene microarrays that allowed the expression of thousands of genes at a time (Clark, 2002; Bertone et al., 2004). However, gene microarrays had technical limitations as they only could analyze known genes, have limited detection of expression signals, false positives because of cross-hybridization between probes and reproducibility issues (Okoniewski and Miller, 2006; Royce et al., 2007).

Since 2008, high-throughput next generation sequencing (NGS) has been used to study the transcriptome. Sequencing the RNA molecules in a cell (RNA-seq) showed to be a powerful tool as it is high-throughput, shows single-base resolution, and doesn’t need the previous knowledge of the genes present in the analyzed genome (Mortazavi et al., 2008; Wang et al., 2009). RNA-seq provides precise measurements for messenger abundance and can distinguish new splicing isoforms and allelic expression. Moreover, new species of non-coding RNAs have been identified by RNA-seq analysis which includes small and long regulatory non-coding RNAs (Reuter et al., 2015; Sahraeian et al., 2017). Among these short RNAs are microRNAs (miRNAs), small interfering RNAs (siRNA), and Piwi−interacting RNAs (piRNAs).

miRNAs are small non-coding RNAs that have emerged as crucial post-transcriptional regulators of gene expression. They are single-stranded non-protein coding RNAs of 20–23 nucleotides that regulate both mRNA stability and translation through direct interaction with the transcripts (Bartel, 2009). miRNA have been shown to be important new players in regulation of stem cell development by playing a critical role in differentiation and maintenance of stem cells (Mathieu and Ruohola-Baker, 2013). Long non-coding RNAs (lncRNAs) have arisen as transcriptional and post-transcriptional regulators, acting at various levels of gene expression. They are defined as non-coding RNAs longer than 200 nucleotides and are present both in the nucleus and cytoplasm of the cell. LncRNAs are involved in proliferation and development through controlling the fate of stem cells, generating a complex network of interactions with regulatory proteins and other RNAs (Ulitsky, 2018; Fico et al., 2019; Xie et al., 2019). Finally, circular RNAs (circRNAs) are a different type of non-coding RNA that can form a covalently closed loop structure and are widely distributed in human tissues and organs. circRNA have also been described as regulators of stem cell fate (Wang et al., 2020).

Here we discuss high-throughput studies, using microarray or RNA-seq results, of MSC induced to adipogenesis, osteogenesis and chondrogenesis. As differentiation processes are highly regulated, initially we will present the variables that may influence the results. Then, we will focus the review in exploring the transcriptome or translatome data indicating key molecules, biological processes, signaling pathways and interaction networks that are essential to induce MSC to an adipogenic, osteogenic or chondrogenic phenotype.

### MSC Differentiation: Important Features to Take Into Account

The analysis of gene expression during differentiation of MSC involves several variables, as stem/stromal source, protocol for stem/stromal cell isolation and for differentiation induction, time point of analysis during the differentiation process and strategy of analysis.

Regarding MSC source, Rebelatto and collaborators have previously described an efficiency of 100% in isolation of bone marrow and adipose tissue-derived MSC. Interestingly, while both had a similar capacity for chondrogenic and osteogenic differentiation, bone marrow-derived MSC produced more mature adipocytes than adipose tissue-derived MSC (Rebelatto et al., 2008). Nevertheless, using donor-matched samples, Mohamed-Ahmed et al. (2018) showed that adipose tissue-derived MSC had a greater adipogenic and delayed osteogenic capacity when compared to bone marrow MSC. Notably, Mohamed-Ahmed et al. (2018) used MSC isolated from young donors (8–14 years) submitted to iliac crest surgery for treatment of cleft lip and palate, while Rebelatto et al. (2008) used bone marrow from the iliac crest from donors with dilated cardiomyopathy (50–70 years) and adipose tissue from donors undergoing elective bariatric surgery and dermolipectomy procedures (26–50 years).

The same kind of tissue could be isolated from different body sites, by different methodologies and from donors with different features. Adipose tissue, for example, may be obtained as residue from several surgery procedures, as liposuction, eyelid plasty treatment, dermolipectomy among others (Table 1). Also, bone marrow may be isolated from iliac crest or metaphysis or proximal diaphysis of the femur (Table 1). Donor’s age may also influence the features of isolated MSC, but, notably, conflicting results have been found regarding the effects of aging. The yield of MSC per volume of tissue was found to be (Ganguly et al., 2019) or not (Ye et al., 2016; Herrmann et al., 2019) affected by aging. Many studies also reported a decrease in the proliferation rate of MSC isolated from older donors (Marêdziak et al., 2016; Ye et al., 2016; Ganguly et al., 2019), while others did not observe age-related differences in the population doubling time (Herrmann et al., 2019). Similarly, aging was also found to decrease (Ye et al., 2016), increase (Marêdziak et al., 2016), or not affect (Zhu et al., 2009) the adipogenic potential of MSC. However, different studies described a reduced osteogenic and chondrogenic potential in MSC from older donors (Zhu et al., 2009; Choudhery et al., 2014; Marêdziak et al., 2016; Ye et al., 2016).

**TABLE 1:** Summary of transcriptome studies that analyzed adipogenic and/or osteogenic differentiation of MSC.

MSC tissue source (surgical procedure, donor age)	Immuno-phenotype (% of positive cells)	Time points	Induction media	RNA type isolated for analysis	Method (platform)	References
**Adipogenesis**						
**Adipose tissue** (liposuction procedure from abdominal subcutaneous adipose tissue; Ages: 19 to 32 years)	CD73+ CD90+ CD105+ CD34- CD45-	1, 7, 14, 21	DMEM; FBS (10%); PEN [10,000 U/mL]/STR [10,000 μg/mL] (2%); DEX (1 μM); IBMX (0.5 M); IND (200 μM); INS (10 μg/mL)	Total RNA	Microarray	Ambele et al., 2016
**Bone marrow** (Ages: NA)	NA	7, 14	MEM-α; FBS (10%); AMPI (100 U/mL); STR (0.1 mg/ml); DEX (1 × 10^–7^ M); IBMX (0.5 mM); IND (50 mM); bFGF (1ng/mL); UltraGlutamine (2 mM)	Total RNA (ST-DGE) and miRNA (RNA-Seq)	ST-DGE and RNA-Seq (Illumina HiSeq 2000)	Casado-Díaz et al., 2017
**Adipose tissue** (bariatric surgery; Ages: 23 to 52 years)	NA	3	hMSC Adipogenic Differentiation Bullet Kit (Lonza)	Total and polysome-associated RNA	RNA-Seq (SOLiD4 System)	Spangenberg et al., 2013; Dallagiovanna et al., 2017
**Adipose tissue** (eyelidplasty treatment; Ages: 20 to 30 years)	NA	1, 7, 14, 21	H-DMEM; FBS (10%); ascorbic acid (5 μg/mL); DEX (1 × 10^–7^ mol/L); IBMX (0.5 mmol/L)	mRNA and miRNA	Microarray	Hu et al., 2018
**Bone marrow** (aspiration from iliac crest; Ages: NA)	CD29+ CD44+ CD105+	3	L-DMEM; FBS (10%); DEX (10^–7^ M); IND (50 ug/ml); IBMX (0.45 mM); ascorbate-2 phosphate (50 μg/ml); INS (0.01 mg/ml)	Total RNA	Microarray	Hung et al., 2004
**Adipose tissue** (liposuction procedure; Ages: 36 to 47 years)	NA	0, 1, 3, 5, 7	Medium 199; FBS (10%); PEN/STR (1%); DEX (1 μM); IND (200 μM); INS (10 μg/mL); methylxanthine (0.5 mM)	Total RNA	RNA-Seq (Illumina HiSeq 2500)	Luan et al., 2015
**Adipose tissue** (bariatric surgery and dermolipectomy procedures; Ages: 33 to 41 years)	NA	3	DMEM-F12; FBS (15%); PEN (100 U/mL); STR (100 μg/mL); DEX (1 μM); IBMX (500 μM); IND (200 μM); INS (1 μg/mL)	Total and polysome-associated RNA (Ribosome Profiling)	RNA-Seq (SOLiD4 System)	Marcon et al., 2017
**Bone marrow** (aspiration from iliac crest; Ages: NA)	CD44+ CD73+ CD90+ CD105+ CD166+ CD14- CD34- CD45-	0, 1, 3, 7, 17	DMEM; glucose (4.5 g/L); FBS (10%); DEX (1μM); IND (0.2 mM); IBMX (0.5 mM); INS (10 μg/ml)	Total RNA	Microarray	Menssen et al., 2011
**Adipose tissue** (subcutaneous adipose tissue from panniculectomy and carotid endarterectomies; Ages: NA)	NA	15	DMEM; FBS (10%); DX (1 μM); IBMX (0.5 mM); INS (1 μg/ml)	Total RNA	RNA-Seq single cell	Min et al., 2019
**Bone marrow** (purchased from BioWhittaker; Ages: NA)	NA	1, 3, 5, 7, 9, 14	H-DMEM; FBS (10%); DEX (1 μM); IND (0.2 mM); IBMX (0.5 mM); INS (0.01 mg/ml)	Total RNA	Microarray	Nakamura et al., 2003
**Bone marrow** (aspiration from iliac crest; Ages: adult donors)	NA	0, 1, 7, 14, 21	MEM-α; FBS (20%); PEN (100 U/ml); STR (100 μg/ml); L-glutamine (2 mM); DEX (0.5 μM); IBMX (0.5 mM); IND (50 μM)	Total RNA	Microarray	Sekiya et al., 2004
**Bone marrow** (aspiration from iliac crest; Ages: NA)	CD44+ CD90+ CD105+ CD19- CD34- CD45-	0, 14	MEM-α; FBS (10%); DEX (1 μM); IBMX (0.5 mM); IND (100 μg/mL); INS (0.01 mg/ml)	Total RNA	Microarray	Xu et al., 2016
**Bone marrow** (Ages: NA)	NA	7, 14, 21, 28	MEM-α; FBS (10%); DEX (1 μM); IBMX (0.5 mM); INS (0.01 mg/ml)	Total RNA	RNA-Seq (Ion)	Yi et al., 2019
**Bone marrow** (Ages: NA)	NA	0, 7, 14, 21, 28	hMSC Basal Medium (Cyagen); DEX (1.0 μM); IBMX (0.5 mM); INS (0.01 mg/ml)	Total RNA	RNA-Seq (Ion)	Yi et al., 2020a
**Bone marrow** (Age: 21 years)	NA	0, 7, 14, 21, 28	hMSC basal medium (Cyagen); DEX (1.0 μM); IBMX (0.5 mM); INS (0.01 mg/ml)	miRNA	RNA-Seq (Ion)	Yi et al., 2020b
**Bone marrow** (Age: 21 years)	NA	0, 7, 14, 21, 28	MEM-α; FBS (10%); DEX (1 μM); IBMX (0.5 mM); INS (0.01 mg/ml)	Total RNA	RNA-Seq (Ion)	Yi et al., 2020c
**Adipose tissue** (stroma-vascular fraction of white adipose tissue from surgical specimens; Age: 4 months).	NA	0, 3, 8	DMEM/Ham’s F12; DEX (1 μM); IBMX (100 μM); INS (0.86 μM); rosiglitazone (1 μM); transferrin (10 μg/ml); triiodothyronine (0.2 nM)	small RNAs	RNA-Seq (SOLiD)	Zaragosi et al., 2011
**Osteogenesis**						
**Bone marrow** (Ages: 34 to 39 years)	NA	Differentiation: 0, ∼10; Mineralization: ∼24	Differentiation: MEM-α; FBS (10%); PEN (100 U/mL); STR (0.1 mg/mL); ascorbic acid-2 phosphate (0.1 M); DEX (10^–8^ M) Mineralization: + BGP (10 mM)	Total RNA (miRNA)	Microarray	Baglìo et al., 2013
**Adipose tissue** (liposuction procedure; Ages: 30 to 55 years)	CD73+ CD90+ CD105+ CD34- CD45- CD133-	0, 28	DMEM; FBS (10%); DEX (0.1 mol/L); BGP (10 mmol/L); ascorbic acid-2-phosphate (50 g/mL)	Total RNA	Microarray	Berdasco et al., 2012; Quan et al., 2016; Zhao et al., 2018
**hBMSC-telomerase reverse transcriptase (TERT) cells**	NA	0, 0.25, 0.5, 1, 3, 7, 10, 13	MEM; FBS (10%); PEN/STR (1%); DEX (10 nM); l-ascorbic acid (0.2 mM); BGP (10mM); 1,25-dihydroxyvitamin D3 (10 mM)	miRNA	RNA-Seq (Illumina HiSeq 2000)	Chang et al., 2018
**Adipose tissue** (plastic surgery; Ages: middle-aged)	CD73+ CD90+ CD105+ CD14- CD45- CD34 (low)	0, 21	DMEM; glucose (4.5 g/L); FCS (10%); antibiotics (1 %); DEX (1 x 10^–7^ M); ascorbic acid (50 μg/mL)	Total RNA	Microarray	Daniunaite et al., 2015; Zhao et al., 2018
**Adipose tissue** (liposuction procedure from sub-abdominal region; Ages: 24 to 68 years) and dental pulp (from deciduous teeth; Ages: 6 to 10 years)	CD29+ (>95%) CD73+ (>95%) CD90+ (>95%) CD105+ (>95%) CD31- (<2%), CD34- (<2%) CD45- (<2%)	0, 4, 6	L-DMEM; FBS (10%); ascorbate-2-phosphate (50 μM); BGP (10 mM); DEX (0.1 μM); PEN (100 U/ml); STR (100 g/ml)	Total RNA	Microarray	Fanganiello et al., 2015
**Bone marrow** (from iliac crest isolated from bone graft surgery; Ages: 19 to 28 years)	CD29+ (>70%) CD44+ (>92%) CD34- (<6%) CD45- (<7%)	14	BGP (10 mM); L-ascorbic acid (50 mM); DEX (100 nM)	Total RNA (miRNA)	Microarray	Gao et al., 2011
**Bone marrow** (from metaphysis and proximal diaphysis of the femur obtained from reconstructive joint surgery; Ages: 46 to 61 years)	Four days after seeding: CD44+ (26%) CD90- CD105- CD166- After confluency and treatment with differentiation medium: CD44+ (>90%)* CD90+ (>90%)* CD105+ (>90%)* CD166+ (>90%)* CD45- (<5%)* CD117- (<5%)*	Differentiation: 0, ∼5.5, ∼10.5, ∼24.2; Mineralization: ∼17.5, ∼23.6, ∼30.7	Differentiation: MEM-α; FBS (10%); PEN (100 U/mL); STR (0.1 mg/mL); ascorbic acid-2 phosphate (100 μM); DEX (10^–8^ M) Mineralization: BGP (10 mM)	Total RNA	Microarray	Granchi et al., 2010
**Periodontal ligament** (from third molars; Ages: 18 to 20 year)	STRO-1+ CD146+ CD31- CD45-	7	DEX (10 nM), BGP (10 mM) and vitamin C (50 μg/ml).	Total RNA (lncRNA, circRNA, mRNA)	RNA-Seq (Illumina HiSeq2000)	Gu et al., 2017
**Bone marrow** (Ages: 67 to 74 years)	NA	1, 3, 7	with or without 10^–7^M DEX	Total RNA	Microarray	Hamidouche et al., 2009; Kang et al., 2019; Yang et al., 2019
**Adipose tissue** (purchased from Cyagen; Ages: 18 to 45 years)	NA	0, 14	OriCell human ASC Osteogenic Differentiation Medium (Cyagen)	Total RNA (lncRNA, mRNA)	Microarray	Huang et al., 2017; Yu et al., 2018; Wu et al., 2018
**Bone marrow** (aspiration from iliac crest; Ages: NA)	CD44+ CD73+ CD105+ CD14- CD19- HLA-DR-	0,14	BGP (10 mM); ascorbic acid (50 mM); DEX (100 nM)	Total RNA	Microarray	Jiang et al., 2019
**Bone marrow** (aspiration from iliac crest; Ages: NA)	CD44+ CD73+ CD90+ CD105+ CD166+ CD11b- CD34- C45- CD117- HLA-DR-	4, 7, 14, 21	DMEM; FBS (10%); PEN (100 U/ml); STR (100 μg/ml); L-glutamine (2 mM); DEX (10 nM); ascorbic-acid-2-phosphate (0.1 mM); BGP (10 mM)	Total RNA	Microarray	Kulterer et al., 2007
**Dental follicle cells** (from third molars; Age: 20 years)	NA	28	MEM-α; FBS (10%); ascorbic acid 2-phosphate (100 μmol/L); KH2PO4 (2.8 mmol/L); DEX sodium phosphate (1 × 10^–7^ mol/l); HEPES (20 mmol/L)	Total RNA	Microarray	Morsczeck et al., 2009
**Periodontal ligament** (Ages: NA)	CD29+ CD44+ CD73+ CD90+ CD105+ CD11b- CD14- CD34- CD45-	4,14	MEM-α; FBS (10%); PEN (100 U/mL); STR (100 mg/mL); L-ascorbic acid phosphate magnesium salt (82 μg/mL); BGP (10 mmol/L); DEX (10 nmol/L)	Total RNA	RNA-Seq (SOLiD System)	Onizuka et al., 2016
**Adipose tissue** (procured from LaCell LLC; Ages: NA)	NA	21	StromaQual; FBS (10%); BGP (10 mM); L-Ascorbic acid 2-phosphate sesquimagnesium salt hydrate (50 μg/ml); DEX (10 nM); 1% antibiotic	Total RNA	RNA-Seq (Ion Proton)	Shaik et al., 2019
**hBMSC-telomerase reverse transcriptase (TERT) cells**	NA	0, 0.25, 0.5, 1, 3, 6, 9,12	MEM; FCS (10%); PEN/STR (1%); BGP (10 mM); L-ascorbic acid (50 μg/mL); DEX (10 nM); calcitriol (1,25-dihydroxyvitamin D3) (10 nM)	Total RNA	RNA-Seq (Illumina HiSeq 2000)	Twine et al., 2014
**Bone marrow** (purchased from Cyagen; Ages: 18 to 20 years)	NA	7	STEMPRO osteogenesis differentiation Kit (Invitrogen)	Total RNA (lncRNA, mRNA)	Microarray	Zhang et al., 2017
**Bone marrow** (purchased from the Shangai Institutes; Ages: NA)	NA	0, 7	MEM-α; FBS (10%); antibiotics (1%); DEX (100 nM); ascorbic acid (0.2 mM); BGP (10 mM)	Total RNA (circRNA, mRNA, miRNA)	Microarray	Zhang M. et al., 2019
**Periodontal ligament** (from premolars; Ages: 12 to 18 years)	CD73+ CD90+ CD105+	0, 3, 7, 14	MEM-α; FBS (10%); PEN/STR (1%); DEX (100 nM); L-ascorbic acid (200 μM); BGP (10 mM)	Total RNA (circRNA, mRNA) and miRNA	RNA-Seq (HiSeq 2000, Illumina)	Zheng et al., 2017
**Chondrogenesis**						
**Bone marrow** (Ages: 38 to 58 years)	NA	21	BMP-2-conditioned medium obtained after incubation of chondrogenic medium [DMEM; DEX (0.1 μM); ascorbic acid (0.17 mM); ITS supplement (1%) (Sigma)] on confluent C9 cells for 48 hours	Total RNA	Microarray	Djouad et al., 2009
**Bone marrow** (from the drill hole of the pedicle during the internal spine fixation; Ages: mean age of 44 years; SD age of 10 years)	NA	3	H-DMEM; FBS (2%); DEX (100 nM); L-ascorbic acid-2 phosphate (50 mM); BD ITS+ Premix (1:100); TGF beta-3 (10 ng/mL)	Total RNA	Microarray	Gong et al., 2018
**Bone marrow** (from iliac crest; Ages: NA)	CD44+ (>97%) CD73+ (>97%) CD90+ (>75%) CD105+ (>93%) CD45- (<1%)	0, 1, 3, 7, 14, 21	H-DMEM; PEN/STR (1%); ITS+ Premix (Corning) (1%); DEX (100 nM); ascorbic acid (50 μg/mL); L-proline (40 μg/ml); recombinant human TGF beta 3 (rhTGF-b3) (10 ng/ml)	Total RNA	RNA-Seq (HiSeq 2500, Illumina)	Huynh et al., 2019
**hMSC** (purchased from Cambrex; Ages: NA)	NA	14	Chondrocyte Differentiation Medium Single Quotes Kit CC-4408 (Cambrex)	Total RNA	Microarray	Ikeda et al., 2007
**Bone marrow** (Ages: NA)	NA	0, 3, 7, 10, 14, 21, 28	H-DMEM; ITS+ (1%); DEX (10^–7^ M); sodium pyruvate (1 mM); ascorbic acid-2 phosphate (120 mM); non-essential amino acids (100 mM); TGF beta-1 (10 ng/mL)	Total RNA	Microarray	Somoza et al., 2018
**Adipogenesis and osteogenesis**						
**Adipose tissue** (liposuction procedure; Ages: 27 to 44 years)	CD73+ CD90+ CD105+ CD11b- CD19- CD31- CD34- CD45- CD117- HLA-DR-	1	(Adi) hMSC Adipogenic Differentiation Medium (hMSC Adipogenic Bullet kit, Lonza) (Ost) hMSC Osteogenic Differentiation Medium (hMSC Osteogenic Bullet kit, Lonza)	Total and polysome-associated RNA	RNA-Seq (Illumina HiSeq 2500)	Marcon et al., 2019, 2020; Robert et al., 2018
**Adipose-derived stem cell-derived cell line**	NA	-2, 0, 0.33, 2, 5, 10, 15	(Adi) DMEM/Ham’s F12; transferrin (10 μg/ml); INS (0.86 μM); triiodothyronine (0.2 nM); DEX (1 μM); IBMX (100 μM); rosiglitazone (100 nM) (Ost) MEM-α; FCS (10%); L-ascorbic acid phosphate (50 μg/ml); BGP (10 mM); DEX (100 nM)	Total RNA	Microarray	Scheideler et al., 2008
**Osteogenesis and chondrogenesis**						
**Bone marrow** (Ages: 33 to 80 years)	CD44+ (100%) CD73+ (100%) CD90+ (>91%) CD105+ (>99%) CD11b- (<1%) CD19- (<1%) CD34- (<1%) CD45- (<1%)	7	(Cho) DMEM; glucose (4.5 g/L); PEN (100 U/mL); STR (100 μg/mL); ITS+ Premix (Corning) (1%); non-essential amino acids (1%); ascorbic acid 2-phosphate (50 μg/mL); DEX (100 nM); TGF beta-1 (10 ng/mL) (Ost) DMEM; glucose (1 g/L); FBS (10%); PEN (100 U/mL); STR (100 μg/mL); ascorbic acid 2-phosphate (50 μg/mL); BGP (5 mM); DEX (10 nM)	Total RNA (RNA-Seq, miRNA and piRNA) and circRNA (Microarray)	RNA-Seq (Illumina NextSeq500) and microarray	Della Bella et al., 2020
**Adipogenesis, osteogenesis and chondrogenesis**						
**Adipose tissue** (from subcutaneous adipose tissue; Ages: NA) and **fibroblasts** (from dermal skin; Ages: NA)	CD73+ CD105+	0, 1, 2, 3, 4, 5, 6, 7	(Adi) DEX (1 μM); IBMX (500 μM); IND (100 μM); INS (10 μg/ml) (Cho) INS (6.25 μg/ml); L-ascorbic acid 2-phosphate (50 μM); TGF beta-1 (10 ng/mL) (Ost) DEX (100 nM); L-ascorbic acid 2-phosphate (50 μM); BGP (10 mM)	Total RNA	RNA-Seq	Jääger et al., 2012
**Bone marrow** (Ages: NA)	NA	0, 1, 3, 7 and 21	(Adi) DMEM-F12; newborn calf serum (5%); DEX (1 μM); IBMX (50 μM); IND (60 μM) (Cho) DMEM; DEX (0.1 μM); ascorbate-2 phosphate (0.17 mM); insulin-transferrin-sodium selenite supplement (1%); TGF beta-3 (10 ng/mL) or BMP-2 (100 ng/mL) (Ost) H-DMEM; FCS (10%); BGP (10 mM); DEX (0.1 μM); ascorbic acid (0.05 mM)	Total RNA	Microarray	Mrugala et al., 2009

*Adi: adipogenesis; AMPI: ampicillin; bFGF: basic fibroblast-growth factor; BGP: β-glycerophosphate; glycerol 2-phosphate; Cho: chondrogenesis; DEX: dexamethasone; DMEM: Dulbecco’s modified Eagles’s medium; FBS: fetal bovine serum; FCS: fetal calf serum; H-DMEM: high glucose – Dulbecco’s modified Eagles’s medium; IBMX: 3−isobutyl−1−methylxanthine; IND: indomethacin; INS: insulin; ITS: Insulin-Transferrin-Selenium; L-DMEM: low glucose – Dulbecco’s modified Eagles’s medium; MEM-α: minimum Eagles’s medium-alpha modification; NA: not available; Ost: osteogenesis; PEN: penicillin; STR: streptomycin; TGF: transforming growth factor.*

Interestingly, several studies using donors from the same group (young or aged, for example) obtained variable results (Zhu et al., 2009; Ganguly et al., 2019; Herrmann et al., 2019) and, stromal cells from different sources were found to be similar upon differentiation (Jääger et al., 2012). The conflicting results and the variability reported suggested that donors characteristics may not be predictive of the specific MSC phenotype (Ganguly et al., 2019; Herrmann et al., 2019). However, these features are certainly important for the analysis and interpretation of the data obtained from different studies; thus, it must always be clearly described in detail in the methodology section. Another difference that may interfere with the results of MSC studies is related to the cell surface markers expressed by the cells. It is recommended to use at least two positive and two negative markers for immunophenotypic characterization of MSC, commonly based on ISCT statements (Bourin et al., 2013). Surprisingly, not all studies shown a complete phenotypic characterization (Table 1). It is important to point out that isolated MSC may have differences in the expression of some markers, which can result in divergences in their proliferation or differentiation potential (Battula et al., 2009; Russell et al., 2010).

For differentiation induction, several protocols have been established to promote adipogenesis, chondrogenesis and osteogenesis which includes the usage of different culture media (Table 1), passage number and confluency. In the manuscripts analyzed in this review, the passage at which differentiation was induced ranged from 2-3 (Kulterer et al., 2007; Gu et al., 2017; Huang et al., 2017; Hu et al., 2018; Shaik et al., 2019) to 15 (Ambele et al., 2016). Regarding confluency, some groups reported that the differentiation was induced with subconfluent cells (up to 90% confluence) (Hu et al., 2018; Yi et al., 2019), others used confluent cultures (Zaragosi et al., 2011; Luan et al., 2015; Xu et al., 2016; Min et al., 2019), or even a few days after the cells reached confluence (Scheideler et al., 2008). Confluency and passaging may affect the differentiation potential of MSC (Wall et al., 2007; Safwani et al., 2014; Abo-Aziza and Zaki, 2017; Noda et al., 2019), although some groups did not find this correlation (Kulterer et al., 2007).

Gene expression may be controlled at different levels by epigenetic, transcriptional, post-transcriptional, translational and post-translational mechanisms. The development of different methodologies allowed the analysis of different aspects of gene regulation during the differentiation process of MSC. One of the most common strategies used is the analysis of total mRNA (Table 1), which yields information about the identity and abundance of mRNA found in different cell types and populations. Then, differences found at the total mRNA level provide information related to the regulation at the transcriptional level and in mRNA stability, though is not always directly equivalent to their translational rate (Tian et al., 2004; Schwanhäusser et al., 2011), and this kind of approach does not contemplate others important aspects of gene expression regulation (Ingolia, 2016).

The mRNAs associated with polysomes and the rate of translation of the transcripts may also be regulated, modulating protein synthesis. The use of methodologies as the polysome profiling (Spangenberg et al., 2013; Marcon et al., 2020) and the ribosome profiling (Ingolia et al., 2009, 2012; Ingolia, 2016; Marcon et al., 2017) are interesting to investigate these aspects of gene expression. The analysis and comparison of total or ribosome free mRNA fraction with the one associated to polysomes or ribosomes may provide important information about the level at which genes are being regulated, identifying mechanisms of translational efficiency (Marcon et al., 2017) and of coordinated/opposite actions of the transcriptional and the post-transcriptional mechanisms (Marcon et al., 2017; Pereira et al., 2019).

Other approaches focused on the analysis of non-coding RNAs, which have an important role in gene expression regulation. These analyses allow the identification of which micro, long non-coding, circular or other non-coding types of RNAs are specifically found in each phase of the differentiation process.

**FIGURE 1:**
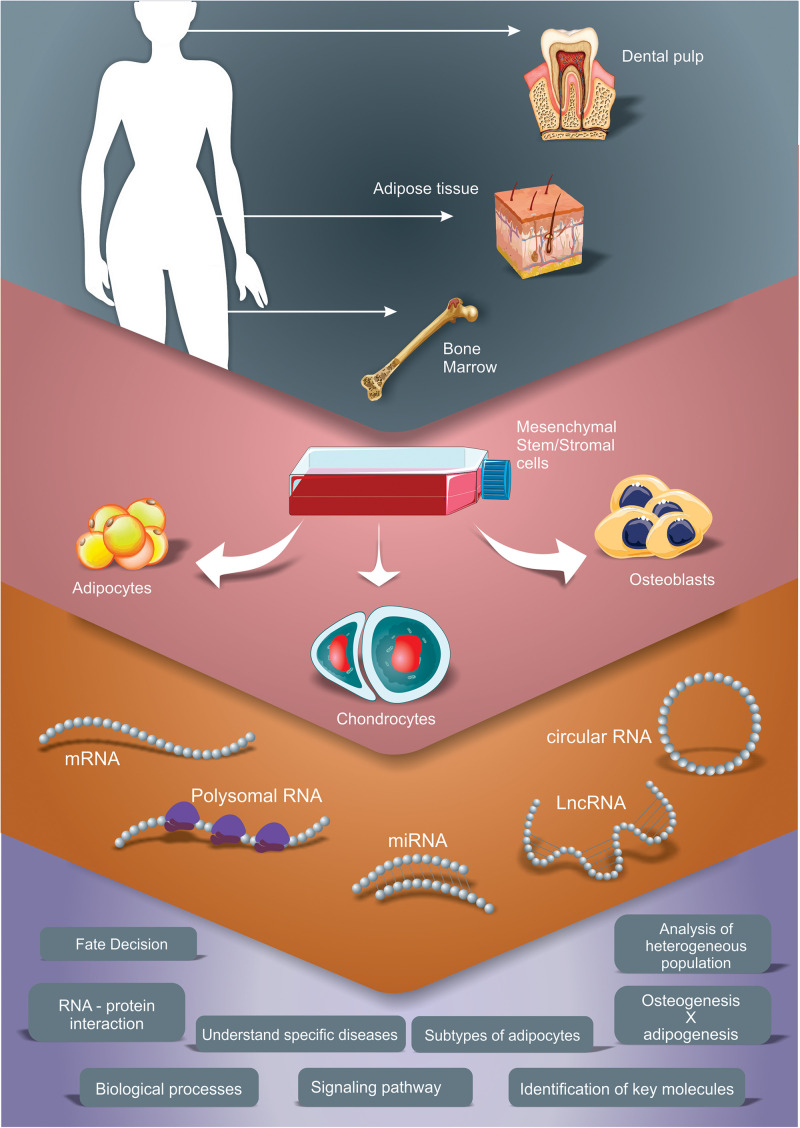
Different transcriptomic approaches to study gene expression profile during adipogenic, chondrogenic and osteogenic differentiation of MSC. The scheme summarizes the studies explored in this review, which used adipose tissue, bone marrow and dental pulp as sources for MSC isolation and performed different induction protocols. Different RNA types were analyzed, as mRNA (by total mRNA, polysome profiling and/or ribosome footprint profiling analysis), microRNA (miRNA), long non-coding RNA (lncRNA) and circular RNA (circRNA). These strategies allow the characterization of main processes, pathways and key factors regulated during the differentiation time course that improve our knowledge regarding osteogenesis, chondrogenesis and adipogenesis.

Following, we will present the findings from studies that used transcriptome analysis to understand the adipogenic, chondrogenic and the osteogenic differentiation process of MSC. Our search was focused in literature that contained results of transcriptomic or gene expression profile studies obtained using high-throughput technologies (microarray, RNAseq) during adipogenesis, osteogenesis or chondrogenesis of human MSC independently from its source. Only studies using *in vitro* differentiation (inductive media) of 2D cultures were considered in this review. Analyzes of various types of RNA, such as mRNAs, miRNAs, lncRNAs and circRNA were contemplated. These studies were summarized in Table 1. By compiling and analyzing these manuscripts, we present some of the main processes, pathways and key factors regulated during the differentiation time course that could improve our knowledge regarding osteogenesis, chondrogenesis and adipogenesis (Figure 1), highlighting the common and the discrepant findings of each group.

## Adipogenesis Vs. Osteogenesis: General Aspects

The differentiation process of MSC both into adipocytes and osteocytes *in vitro* takes about 20 days to be accomplished, and can be divided in two main steps: lineage commitment – from MSC to a committed progenitor – and maturation – from progenitors to mature phenotypes (Chen et al., 2016). After the beginning of adipogenic induction, AMP cyclic production is augmented, leading to the phosphorylation of CREB. Transcriptional factors CEBPB and D are also upregulated in the 1st hours of adipogenic treatment. Once activated by phosphorylation, CEBPB binds to regulatory elements and stimulates CEBPA and PPARG transcription. While CEBPB synthesis decays, CEBPA and PPARG transcription is continuously stimulated by CEBPA binding to CEBPs regulatory elements. PPARG transcription is also controlled by transcription factors SREBP1 and KLF family (reviewed by Rosen and MacDougald, 2006; Chen et al., 2016). The later stages of adipogenic differentiation are marked by the expression of fatty acid synthase (FAS), glycerophosphate dehydrogenase, acetyl CoA carboxylase, malic enzyme, glucose transporter type 4 (Glu4), insulin receptor and adipocyte-selective fatty acid binding protein (aP2), and by the formation of lipid, which are characteristic of adipocytes (reviewed by Rosen and Spiegelman, 2000).

During early osteogenesis, there is an upregulation of hedgehog proteins, Wnt/β-catenin signaling, BMPs and endocrine hormones, besides epigenetic regulators and growth factors. Then, one of the key factors involved in the osteogenic differentiation, RUNX2, is upregulated. Besides stimulating osteogenesis, RUNX2 also inhibits adipogenesis. But while RUNX2 expression decays along the differentiation process, Osterix and β-catenin upregulation is kept and is important to the maturation of osteoblasts (reviewed by Chen et al., 2016; Pierce et al., 2019). Alkaline phosphatase, osteoprotegerin and type I collagen are also expressed in more advanced stages of osteogenesis, while osteocalcin is related to the terminal differentiation phase (reviewed by Pierce et al., 2019).

One of the main questions about the differentiation process is at what moment during the differentiation time course MSC become committed to a specific phenotype. In our previous works, we observed that, in the first 24 h of osteogenesis of MSC, key genes related to the osteogenic differentiation were not differentially expressed, notwithstanding we found another set of differentially expressed genes related to ossification and bone mineralization (Robert et al., 2018). Conversely, in MSC treated with adipogenic medium for 24 h, adipogenesis related genes were already differentially expressed, including transcription factors and genes related to lipid metabolism (Marcon et al., 2019), which will be discussed later in this work. Although differentiation genes were already regulated in the first 24 h of induction, the MSC were still not committed with a specific lineage (Spangenberg et al., 2013).

Jääger et al. (2012) compared the gene expression profile of MSC and dermal fibroblasts during adipogenic, osteogenic and chondrogenic differentiation processes over 7 days of differentiation. PCA analysis of the differentially expressed genes suggested that the switch of stromal cell regulatory mechanisms into phenotype-specific regulation happens earlier in adipogenesis than in osteo and chondrogenesis (Jääger et al., 2012). Ambele et al. (2016) suggested that the commitment with the adipogenic lineage only happens after day 7 of induction. On the other hand, Scheideler et al. (2008) analyzed gene expression in adipogenic and osteogenic-induced MSC in different timepoints (8 h, 2, 5, 10 and 15 days of induction), and suggested that lineage commitment to adipogenic or to osteogenic phenotype happens between 2 and 5 days of induction. During this period, they identified 39 genes that were upregulated in MSC induced to osteogenesis and downregulated in adipogenesis induced MSC. Interestingly, 5 of these genes contained binding sites to SREBP. On the other hand, 26 genes were specifically upregulated in adipogenesis and downregulated in osteogenesis, including transcripts containing PPARGDR1 and LXR response elements (Scheideler et al., 2008). These different studies suggest that during early adipogenesis (up to 48 h), MSC already triggered physiological changes that led to changes in gene expression but are still not committed with a lineage specific phenotype. This commitment happens around 2 to 7 days of differentiation, and the variability found may be related to different methodologies used for MSC isolation and induction media/protocol (Table 1).

Beside the regulation at the total mRNA level, gene expression during early adipogenesis and osteogenesis is also regulated by controlling the association of transcripts with the translational machinery. This observation has been consistent in studies using different induction times and methodologies for analysis. The comparison of the total and the polysome associated mRNA analysis demonstrated that more genes were identified as differentially expressed in the polysomal fraction during early osteogenesis (Robert et al., 2018). A similar pattern was observed in the first 24 h of adipogenesis (Marcon et al., 2019). On the other hand, after 72 h of adipogenic induction different results were obtained. More genes were identified as differentially expressed in the total fraction than in the polysomal using polysome profiling (Spangenberg et al., 2013). Conversely, using the ribosome profiling methodology more DEG were found in the fraction associated with ribosomes (Marcon et al., 2017). The differences observed in these two studies may be related to differences in the source of MSC (adipose tissue from obese donors underwent to bariatric surgery vs. healthy donors submitted to liposuction surgery) or in the experimental methodology (polysome vs. ribosome profiling) (Table 1). Nevertheless, in all the analysis performed, it was demonstrated that the association of mRNAs with the translational machinery is an important step for the regulation of gene expression during the adipogenic and osteogenic differentiation process of MSC.

## Gene Expression Profile in Adipogenesis of MSC

Initially, the study of the adipogenesis process focused on murine models, such as 3T3-L1 cells (reviewed by Basu et al., 2013; Ruiz-Ojeda et al., 2016). Nakamura et al. (2003) analyzed human MSC induced to adipogenesis for 0, 1, 3, 5, 7, 9 and 14 days. It was found 197 genes modulated over differentiation, with the higher number of upregulated genes on days 3, 7 and 9. Cluster analysis showed that the downregulated genes included markers from other lineages, cytoskeleton and extracellular matrix (ECM). On the other hand, other clusters could be divided into 2 groups: one with genes that were involved in the early stage (days 0–6) and another with genes related to a later stage (days 7–14) of adipogenesis (Nakamura et al., 2003). Among the upregulated genes at early stages were identified CEBPB and D, SWI/SNF complex (BAF60b) and transcription factors as SLUG, FKHR; at late stage they found CEBPB and D, mitogen-activated protein kinases, CDC2-associated protein, cycline G1, PPARG, CEBPA, FABP-a, LPL and others related to lipid metabolism and adipocyte differentiation (Nakamura et al., 2003). That study, according to the authors, was the first that identified genes related to early stages of adipogenesis, using MSC.

Other gene expression profile analyses during adipogenesis identified several upregulated genes related to metabolism (as gluconeogenesis, fatty acid synthesis), secreted proteins, as apolipoprotein E, TGFb, IGF1 and 2 and seven transcription factors with marker time-dependent increase: ZEB (day 1), ZNF145 (day 7) PPARG (day 14), c-fos (day 14), SOX4 (day 21), CEBPA (day 21), and Forkhead (day 21) (Sekiya et al., 2004). The identification of DEG at 3-day adipogenic induced MSC showed 82 and 31 up and downregulated genes, respectively, many of which had not yet been described as related to the adipogenesis process. Genes related to growth arrest and lipid metabolism (as APOD, PPAP2B, CES2) were among the upregulated ones, while those related to other differentiation lineages, as neural, epithelial or osteogenic, had reduced expression at this time point (Hung et al., 2004). These results indicated an early change in gene expression profile of MSC that were induced to an adipocyte phenotype.

In fact, different groups have demonstrated that in the 1st days of adipogenic induction, the MSC are still not committed with the adipogenic phenotype (Scheideler et al., 2008; Spangenberg et al., 2013), but already have a significant change in the gene expression profile (Scheideler et al., 2008; Spangenberg et al., 2013; Ambele et al., 2016; Marcon et al., 2019). Transcriptomic analysis confirmed the upregulation of key transcriptional factors during early adipogenesis. Different analysis demonstrated that KLF15 expression was detected in the first 24 h of induction (Ambele et al., 2016; Marcon et al., 2019, 2020), and remained upregulated after 3 (Spangenberg et al., 2013), 7, 14 and 21 (Ambele et al., 2016) days of adipogenic treatment. An augmentation of LMO3, FOXO1, ZBTB16 (Ambele et al., 2016; Marcon et al., 2019) CEBPB and CEBPD (Marcon et al., 2019) mRNA was also detected in the total and in the polysome-associated mRNA fraction (Marcon et al., 2019), suggesting not only an upregulation in terms of mRNA abundance but also in protein synthesis rate.

In the first 24 h of adipogenic differentiation of MSC, it was also demonstrated that the genes related to cell cycle and proliferation were mostly downregulated, and this scenario was accompanied by a decrease in cell proliferation and G1-cell cycle arrest. Interestingly, the downregulation of these transcripts was observed both in the total and in the polysome associated fractions, suggesting that the expression control is mainly promoted by the control of mRNA abundance (Marcon et al., 2019).

After 3 days of adipogenic treatment, genes related to lipid metabolism and adipogenesis are upregulated (Spangenberg et al., 2013; Luan et al., 2015; Marcon et al., 2017). Different studies reported the upregulation of the key transcription factors involved in adipogenesis, PPARG and CEBPA, at this time point (Spangenberg et al., 2013; Luan et al., 2015), but others have reported that those genes were only upregulated after 14 days of adipogenic treatment (Ambele et al., 2016).

In 14 days of adipogenesis, the primary most significant GO categories for upregulated genes were collagen fibril organization, brown fat cell differentiation, and positive regulation of fat cell differentiation (Xu et al., 2016). The main GO categories for downregulated genes were cell cycle, S phase of mitotic cell cycle, and G1/S transition of mitotic cell cycle (Xu et al., 2016), also observed in 24 h by previous studies (Marcon et al., 2019). Notably, it is possible to observe that the inhibition of genes related to cell proliferation or cell cycle were observed at several differentiation time points, indicating a stop of proliferation throughout the commitment to adipocytes.

Investigating the expression profile of mRNA and miRNA, after 7 and 14 days of adipogenesis, Casado-Díaz et al. (2017) identified more than 2000 and 100 regulated mRNAs and miRNAs, respectively. These genes were related to different pathways, including PPARG, lipid, carbohydrate and energy metabolism, redox, membrane-organelle biosynthesis, and endocrine system. The adipogenesis upregulated-genes were clustered into five groups: metabolism, response to stimulus, cell differentiation, biological regulation, and lipid storage. This indicated a relevant activation of cellular metabolism during adipogenesis. On the other hand, the downregulated genes were clustered into seven groups: developmental process, movement and transport, regulation of cellular process, apoptotic process, response to stimulus, cell adhesion and organization of cellular components (Casado-Díaz et al., 2017). Probably cytoskeleton reorganization during adipogenesis might affect survival, adhesion capacity and cell shape. Complementary analysis of mRNA-miRNA interaction showed that repressed miRNA-encoding genes can act downregulating PPARG-related genes, mostly the PPARG activator (PPARGC1A) (Casado-Díaz et al., 2017).

Characterization of early and late stages of adipogenesis showed 85 upregulated genes which were involved in PPARG signaling pathway (RXRA, CEBPA, CES1, PPARG, GPD1), “adipocytokine signaling pathway” (LPL, ADIPOQ, APOE, LGALS3, COL5A3, APOL6, CLEC1A, FLRT3), “adipocyte differentiation pathway” (FABP4, PLIN) and others. Also, they highlighted some other genes that could regulate adipogenesis, as SCARA5 and MRAP (Menssen et al., 2011).

Looking for key genes that could regulate adipogenesis, a temporal gene expression analysis was performed evaluating MSC induced for 7, 14, 21 and 28 days with adipogenic medium (Yi et al., 2019). Among the DEGs, 20 were identified with potential key genes responsible for adipogenesis: the upregulated PPARGC1A, ACACB, ACSL1, FABP4, FASN, IGF1, IRS2, LEP, LEPR, LIPE, PCK1, PDE3B, PLIN1, SCD, SOCS3, STAT3; and the downregulated BDNF, F2R, RAC2, RAPGEF3 (Yi et al., 2019). Previous study, from Casado-Díaz et al. (2017) found the following top upregulated genes during adipogenesis (7 and 14 days): LPL, FABP4, TIMP4, ADIPOQ, PLIN1, AOC3, PPP1R1A and ADH1B. The downregulated genes included some associated to MSC differentiation into osteoblasts as chitinase-3-like (CHI3L1), biglycan (BGN) and “four and a half LIM domains 2” (FHL2) (Casado-Díaz et al., 2017). Interestingly, only PLIN1 and FABP4 were common between both studies. Although both studies used bone marrow MSC and the differentiation medium were very similar, the differences may be due to the presentation of the data: while one indicated the top genes more or less expressed in 7–14 days (Casado-Díaz et al., 2017), the other identified those common in a period of 7-14-21-28 days of differentiation and still appeared in GOs related to adipogenesis (Yi et al., 2019).

GO terms related to metabolism were represented in many analyses of adipogenesis data. Whole transcriptional profiling of cellular metabolism during adipogenesis from MSC explored signaling pathways and metabolism of glucose, amino acid and fatty acid. It was shown that metabolism related pathways and the PI3K−Akt signaling pathway were the most enriched pathways using GO analysis (Yi et al., 2020b). The PI3K−Akt signaling pathway stimulated and directly regulated cellular metabolism by targeting the potential key genes, such as FASN, PCK1, SCD, and SLC2A1 and priming glucose aerobic glycolysis, arginine and proline metabolism, glutathione metabolism, and arachidonic acid metabolism during adipogenesis (Yi et al., 2020b). Also, analyzing polysomal RNA obtained after 3 days of adipogenic induction, it was indicated a change in the energetic profile in induced compared to non-induced cells (Drehmer et al., 2016). The reactive oxygen species (ROS) production, for example, was increased after 3 days of adipogenesis and could be involved in the differentiation process (Drehmer et al., 2016).

Considering the complexity of the gene expression regulation in diverse biological processes, including differentiation, the identification of small and non-coding RNAs that are differentially expressed during adipogenesis of MSC were also performed. Zaragosi et al. (2011) identified twenty-one miRNAs that were upregulated during differentiation, while five miRNAs were downregulated using deep sequencing. This approach revealed the un-annotated miR-642a-3p as a highly adipocyte-specific miRNA. Inhibition of the miR-30 family blocked adipogenesis, whilst over-expression of miR-30a and miR-30d stimulated this process. One of the miR-30 target is the RUNX2 (osteogenesis transcription factor) which could be, at least in part, responsible for miR-30 positive effects on adipocyte differentiation (Zaragosi et al., 2011). Another study using microarray analysis found 32 miRNAs differentially expressed during adipogenesis, among them is miR27b which was downregulated, while lipoprotein lipase (LPL) mRNA was up-regulated (Hu et al., 2018). miR-27b targeted LPL and inhibits adipogenic differentiation (Hu et al., 2018).

A miRNA expression profile performed by Yi et al. (2020a) found a total of 39, 105, 194, and 112 differentially expressed miRNA at 7, 14, 21, and 28 days of adipogenesis, respectively (Yi et al., 2020a). Among the 25 most significant miRNAs, the majority (14) were upregulated. Of these, nineteen miRNAs potentially targeted for 35 mRNA that were associated, e.g., with lipid droplets formation. Among the identified miRNAs, five were highlighted, including hsa−miR−146a−3p, hsa−miR−4495, hsa−miR−4663, hsa−miR−6069, and hsa−miR−675−3p that could be regulating adipogenesis, once its targets were ACSL1, APOB, METTL7A, PLIN1, and PLIN4, and were potentially involved in lipid droplets formation (Yi et al., 2020a).

Luan et al. (2015) identified 2868 transcripts differentially expressed over days 0, 1, 3, 5, and 7 of adipogenic differentiation of MSC. As expected, the upregulated ones had relation with adipocyte differentiation and function, while de genes downregulated were more related to regulation of cell cycle. Moreover, they found 207 lncRNAs differentially expressed (109 up and 98 downregulated). A “guilt-by-association” analysis pointed out that 26 lncRNAs, all upregulated, assigned for fat-related GO terms, including HSD17B7P2, AQP7P1 and AQP7P3 (Luan et al., 2015).

It was previously demonstrated that a high percentage of lncRNAs are actively mobilized to or from polysomes during early stages of adipogenesis (Dallagiovanna et al., 2017). Non-coding RNAs can also be regulators of gene expression by forming complexes with proteins and other types of RNAs, including mRNAs and miRNAs (Guttman and Rinn, 2012). Dallagiovanna et al. (2017) analyzed the lncRNAs associated with polysomes and identified a great number of lncRNAs regulated in this RNA fraction. Among the differentially expressed lncRNAs, there are pathways related to cell growth and proliferation and a network formed by H19 (*gene* for a long non-coding RNA) interaction partner. Besides that, 43 lncRNAs targeted miRNAs, of which 16 were previously described as having a relevant role in adipogenesis. Between them is lncRNAmir22HG with several binding sites to the miR-30 family and which was more abundant in the control compared to induction (Dallagiovanna et al., 2017). Once that during adipogenesis, lncRNAmir22HG is less abundant, the concentration of those miRNAs might be higher and stimulate the differentiation. Interestingly, previous work had shown that the reduction of mir-30 reduced adipogenesis (Zaragosi et al., 2011). Differences in the analyzed time points and RNAs can generate results that seem conflicting, otherwise indicating the complexity of gene networks that could govern the commitment to the adipogenic lineage.

Yi et al. (2020c) reports global transcriptional profiling of alternative splicing events during adipogenesis from MSC by transcriptome technique. Among the identified 122 alternative splicing events, the three genes including actinin alpha 1 (ACTN1), LDL receptor−related protein 1 (LRP1), and latent transforming growth factor beta binding protein 4 (LTBP4), appeared in multiple alternative splicing types at 7, 14, 21, and 28 days (Yi et al., 2020c). Moreover, the differentially expressed genes displayed changes in the length of their 3′untranslated regions (3′UTR) during the adipogenesis (Spangenberg et al., 2013). The splicing events and changes in UTR regions may be associated with the ability to associate with ribosome or in mRNA half-life.

Mesenchymal stem/stromal cells are heterogeneous with respect to phenotype and function in current isolation and cultivation regimes, which often lead to incomparable experimental results (Mo et al., 2016). The study of transcriptomes of cell populations derived from single MSC before and after adipogenic differentiation and before and after thermogenic activation allowed the identification of a minimum of 4 distinct human adipocyte subtypes that can differentiate from mesenchymal progenitor cells (Min et al., 2019). The new technologies and studies of single cells will be able to expand our knowledge about the different subpopulations of MSC.

## Gene Expression Profile in Osteogenesis of MSC

The potential of MSC as a therapeutic alternative for bone regeneration led to an attempt to understand the process of osteogenesis *in vitro*. Many of the pathways and molecules involved - and used as differentiation markers - are known (reviewed by James, 2013; Rutkovskiy et al., 2016; Wu et al., 2016), but the complete process of osteogenic commitment is not yet fully understood. Thus, many studies focused on using transcriptome analysis to understand the molecular events, gene expression profiles and post-transcriptional regulation, that are essential for each stage of the MSC osteogenic differentiation process.

The presence of previously known factors related to osteogenesis, such as RUNX2, OCN, ALPL, was confirmed in several studies using transcriptomic analysis (Twine et al., 2014; Shaik et al., 2019). However, depending on the time point analyzed, the classical markers did not appear. For example, the study that evaluated the modulation of gene expression using polysomal mRNA analysis during the first 24 h of osteogenic induction did not find markers of osteoblast commitment, such as RUNX2 and BMP4. On the other hand, it presented other factors related to the ossification process such as BMP6, Forkhead box O1 (FOXO1), osteomodulin (OMD) among others that could have important functions at initial stages of osteogenesis (Robert et al., 2018). Other studies have shown that the expression of RUNX2 and ALPL, for example, appears at later moments of osteogenic differentiation (Twine et al., 2014), but no regulation was observed after 28 differentiation induction days (Morsczeck et al., 2009).

Granchi et al. (2010) characterized early, intermediate and late stages of osteogenic differentiation in an osteogenic differentiation protocol with two main stages: MSC differentiation (days 5, 10, 24) and MSC mineralization (days 18, 24, 30) (Table 1). The main differences were verified at final stages of MSC differentiation and mineralization. Considering the genes upregulated throughout the process, most of them belonged to pathways and GOs related to bone cell biology. Specifically, the upregulated genes at final MSC differentiation were more related to cell communication which involved growth factors and adhesion genes. Genes related to angiogenesis appear in all analyzed time points, but its proportion is higher at initial mineralization process (Granchi et al., 2010). The group also indicated some genes to use as osteogenic markers as ANKH, COMP, DKK1, DKK3, FGF2, ICAM1, SOX9, SPOCK1, and TIMP3 (Granchi et al., 2010).

Another temporal expression profile of mRNAs and miRNAs differentially expressed during osteogenesis were performed comparing eight time points: 0, 6, 12, and 24 h, and 3, 6/7, 9/10, and 12/13 days post-osteogenic differentiation induction (Table 1; Twine et al., 2014; Chang et al., 2018). In general, the process was divided into stages including: an early stage, regulating the cell proliferation, an intermediate stage related to the commitment of cell to osteoblasts and matrix maturation, and finally reaching the late stage of matrix mineralization (Chang et al., 2018). These osteogenic stages were also characterized in previous studies, as one that evaluated the regulated mRNAs during 4 time points of the differentiation process, indicating 3 genes, ID4, CRYAB, and SORT1, with potential influence on osteogenesis, and describing the activation of Smad pathways induced by BMPs, TGFb and inhibin in the process (Kulterer et al., 2007).

Looking for genes associated with osteoblast phenotype, Twine et al. (2014) found 332 skeletal related genes in its mRNA dataset. Of these, it was selected 123 genes that could be recognized as markers of osteogenic differentiation. Most of the selected markers were related to secreted proteins and extracellular matrix (more than 50%). In addition, genes with peak expression at the beginning of differentiation (0–24 h) and in the intermediate stage (3–6 days) are related to ECM organization, skeletal system development and processes involved in cellular adhesion. On the other hand, late stages of osteogenesis (9–12 days) were enriched in genes related to osteoblast differentiation, cell migration and others (Twine et al., 2014). Interestingly among the genes with higher counts per million (cpm) values it was found fibronectin (FN1), COL1A1, COL1A2, COL6A3, THBS1 and SPARC, all related to extracellular space or secreted proteins (Twine et al., 2014).

Another possibility for analysis using transcriptome data is the development of interaction networks, not only between genes, but also between GOs and pathway analysis. Recent work has shown more downregulated than upregulated genes after 14 days of osteogenic induction of bone marrow derived-MSC (Jiang et al., 2019). The biological processes and pathways that have been highlighted among downregulated genes were mainly involved in cell proliferation and cell cycle, while extracellular matrix organization and interaction, cell adhesion, complement and coagulation cascades and ossification are highlights among upregulated ones. The pathway network demonstrated that during differentiation there was, for example, interaction between “Focal adhesion” with “ECM-receptor interaction,” “Regulation of actin cytoskeleton,” and “Cell cycle” pathways (Jiang et al., 2019). Interestingly, biological processes related to cell adhesion, proliferation and communication have also been identified after only 24 h of differentiation (Robert et al., 2018). This shows, along with other studies, the complexity of signaling necessary for an efficient osteogenic differentiation process.

One of the GOs that is common in many of transcriptome analysis during MSC osteogenesis were related to extracellular organization, cell communication and adhesion, both in early and late stages. Recently Shaik et al. (2019) focused on exploring the data related to ECM differentially expressed genes identified after 21 days of osteogenesis. In order to investigate the possible role of ECM and secreted proteins during bone formation and angiogenesis, the group compared their results with matrisome data (Hynes and Naba, 2012; Hynes, 2014) and observed that a great number of glycoproteins, secreted factors, ECM-affiliate genes were upregulated at late stage of osteogenesis. On the other hand, ECM remodeling enzymes, as MMPs and ADAMTS were more downregulated, while many subunits of integrins (ITGA10, 4) showed increased expression compared to undifferentiated MSC (Shaik et al., 2019). Regarding integrin expression and osteogenesis, Hamidouche et al. (2009), showed that the integrin α5 subunit (ITGA5), different from that observed by others (Shaik et al., 2019), is upregulated during osteogenesis and its expression was sufficient to promote osteogenic differentiation (Hamidouche et al., 2009).

The increased presence of secreted pro angiogenic factors, including CXC cytokines, after osteogenesis was another interesting finding (Shaik et al., 2019), indicating the possible regulation of bone development together with the development of new vessels. DEG during only 24 h of osteogenic induction already indicated a process related to vasculature development, GO also found in genes that have high expression after 24 h and 12 days, where VEGF was an example (Twine et al., 2014). The proliferation-related GO is also very common to appear among osteogenesis regulated genes, but while some studies indicate a stop in the cell cycle (Chang et al., 2018; Jiang et al., 2019), others report an increase in proliferation (Robert et al., 2018).

In addition to the possibility of identifying GOs and pathways, there are also studies that focus on identifying transcription factors and/or verifying interaction between genes or proteins, looking for those who have a central role in networks. Analysis of DEG identified over 1, 3 and 7 days of osteogenic differentiation identified some transcription factors as central nodes in interaction networks such as FOS, SOX9, EP300, CREBBP, ESR1 and EGR1 (Kang et al., 2019). Others, based on data from MSC differentiation to osteoblasts during 28 days (Berdasco et al., 2012) showed interesting protein-protein interaction with nodes as VEGFA, IL1B, EDN1, FG2 and others, some of them shared with myogenic induced MSC (Quan et al., 2016).

The osteogenic differentiation potential varies depending on the MSC source. Fanganiello et al. (2015) compared the efficiency of dental pulp (deciduous teeth) derived- MSC (hDPMSC) with adipose tissue derived-MSC (hASC) in promote osteogenic differentiation, using bone marrow MSC (hBMMSC) as control (Fanganiello et al., 2015). Transcriptomic analysis was performed after 4 and 6 days of osteogenesis induction and it was shown that the largest number of regulated genes was found in differentiated cells derived from bone marrow, while the smallest was in hDPMSC. Comparing the data, 11 DEGs were common between hDPMSC and hBMMSC, which were related to osteogenic pathways, but do not appear in hASC. On the other hand, 47 DEG were shared between hBMMSC and hASC, generating pathways such as serine biosynthesis (Fanganiello et al., 2015). Additionally, despite hASC and hDPMSC express osteogenic markers the gene expression was higher in dental derived cells, which showed greater potential for osteogenesis. Looking for markers that could indicate cells with best osteogenic potential, IGF2 and ITGA8 were highlighted considering that both had higher expression over osteogenesis in hDPMSC compared to hASC. Specifically, cells showed more ALP activity and matrix mineralization when IGF2 level was higher. This could indicate that cells with higher levels of IGF2 before the beginning of osteogenic differentiation were more predisposed to the osteogenic phenotype (Fanganiello et al., 2015).

Also, regarding tooth-derived cells, analysis of the transcriptome of dental follicle cells induced to osteogenesis identified that 98 genes were upregulated in the process and were related to extracellular space and immune response (Morsczeck et al., 2009). Interestingly, downregulated genes were also composed of extracellular space proteins, profile also observed in other studies (Shaik et al., 2019). IGF-2, CD14 and transcription factors as KLF9, NR4A3, PRDM1, ALF, TSC22D3 and ZBTB16 were some of the identified upregulated genes in cells induced to osteogenesis (Morsczeck et al., 2009). The zinc finger and BTB domain containing 16 (ZBTB16) was also identified in other data from periodontal ligament-derived MSC differentiated to osteoblasts. It was shown that this zinc finger had increased expression during osteogenesis and its silencing decreased the expression of osteogenic markers (OCN and BSP) and ALP activity (Onizuka et al., 2016). Results from Osx knockdown indicated that expression of ZBTN16 depended of Osx; and, chromatin immunoprecipitation assay also indicated that Osx is an upstream regulator of ZBTB16 (Onizuka et al., 2016).

Considering the differences presented between transcriptomic datasets, joint analysis of different data could result in relevant information for the understanding of osteogenesis. Comparison of datasets could allow the identification of key genes presented in different osteogenic differentiation protocols (Yang et al., 2019) which may indicate an essential role in the differentiation process. Beside that, re-analysis of microarray data from osteogenic differentiated adipose derived-MSC (Berdasco et al., 2012; Daniunaite et al., 2015) showed that 142 and 69 genes were up and downregulated, respectively, in both datasets (Zhao et al., 2018). GO and pathway analysis of DEG indicated that they were enriched in terms associated with “ECM organization,” “angiogenesis,” “Wnt protein binding,” “FXR/RXR activation,” and “adipogenesis pathway,” among others. Protein-protein interaction network highlighted central nodes composed of, e.g., FOXO1, ID2, IL1B, NID1, PER1, LGR4, STK32B. The reduction of FOXO1, in fact, was shown to be able to reduce expression levels of osteogenic markers and the amount of calcium nodules (Zhao et al., 2018). Thus, FOXO1, identified after 24 h (Robert et al., 2018) and at later stages of osteogenesis (Zhao et al., 2018) could be an important regulator of osteogenesis.

In addition to the mRNA analysis, great interest has arisen trying to understand how the transcribed RNAs could be regulated. Thus, identification of miRNAs, lncRNAs and also circRNAs that are essential to the process allow advances in the understanding of post-transcriptional regulation occurring in the differentiation process.

There are several reports that point out the influence of different miRNAs in regulating pathways related to osteogenesis (reviewed by Martin et al., 2016; Li, 2018). Using 3 MSC donors, a study showed that there is a difference in the differential expression of miRNAs during osteogenesis among the donors: while one of them had more than 50 differentially expressed miRNAs, the other one showed less than 30 (Gao et al., 2011). Considering those that appeared in at least two donors, it was found 8 downregulated (hsa-miR-31a, hsa-miR-106a, hsa-miR-148a, hsa-miR-424, hsa-miR-210, hsa-let-7i, PREDICTED_MIR191, hsa-miR-99a) and 5 upregulated (hsa-miR-30a-5p, hsa-miR-30c, hsa-miR-130a, hsa-miR-15b, hsa-miR-130b) miRNAs (Gao et al., 2011). Some of these miRNAs, e.g., members of the let-7 family and miR-31, have also been reported in other studies, although not always with the same expression profile (Baglìo et al., 2013; Chang et al., 2018).

A miRNA expression profiling analysis during osteogenesis found 29 and 5 miRNAs modulated during differentiation and mineralization stages, respectively (Baglìo et al., 2013). Among the upregulated miRNA, miR-31, miR-145, and miR-504 appear to have potential to regulate Osterix (by binding to the 3’UTR), a known transcriptional factor involved in osteogenesis (Sinha and Zhou, 2013). Indeed, it was demonstrated that the reduction of miR-31 increased Osterix expression, indicating that miR-31 is a regulator of Osterix (Baglìo et al., 2013). Besides that, reduction in miR-31 expression also positively affects expression of RUNX2 and BMPR2 (Gao et al., 2011), indicating that miR-31 could be an important regulator of osteogenesis.

The expression profile analysis of miRNAs during 8 time-points of osteogenic differentiation showed that of the 204 miRNAs filtered, 31 were selected to verify its influence in osteogenesis (Chang et al., 2018). Nineteen showed a decrease in ALPL activity when overexpressed, highlighting the negative effect in osteogenesis of, e.g., miR-512, miR-146a and miR-146b, miR-320a, miR-210, miR-222, miR-423, and miR-138 (Chang et al., 2018).

An interesting approach is to evaluate the potential targets of these miRNAs. miR-30, for example, which is upregulated in differentiation (Gao et al., 2011), has among its targets the CXCL12 (or SDF1), gene that has already been described as having decreased expression in the osteogenesis process (Morsczeck et al., 2009). Another example was the miR-15b, identified as overexpressed by Gao et al. (2011), and with decreased expression during osteogenesis by Chang et al. (2018). Among the targets of miR-15b is the FGF2 (Gao et al., 2011). The expression levels of this gene showed different profiles in different studies: while some showed a decrease in its expression (Morsczeck et al., 2009; Shaik et al., 2019), other indicated that FGF2 increase over osteogenesis (Granchi et al., 2010) or had a bimodal expression, being overexpressed at the beginning and at the end of differentiation process (Twine et al., 2014). These studies, additionally to the variation in the analyzed time points, presented differences in the methodology for RNAs identification and in the differentiation protocols (Table 1). However, all these generated data can be analyzed together, enabling the formation of a complete network.

The important role of lncRNAs in regulating osteogenesis, or even in bone-related diseases, has been explored over the years (reviewed by Silva et al., 2019; Zhang J. et al., 2019). When evaluating gene expression profile of MSC after 7 days of osteogenic induction more than 1200 mRNAs and lncRNAs were differentially expressed in relation to undifferentiated cells, with the majority being upregulated (Zhang et al., 2017). Among the processes and pathways highlighted using mRNA analysis it was shown: response to stimulus, DNA-dependent transcription, cell adhesion, skeletal system development, cytokine-cytokine receptor interaction, ECM-receptor interaction and others. In addition, interaction analysis of mRNAs and lncRNAs identified seven mRNAs (GPX3, TLR2, BDKRB1, FBXO5, BRCA1, MAP3K8, and SCARB1) and six lncRNAs (XR_111050, NR_024031, FR374455, FR401275, FR406817, and FR148647) that could be regulatory genes. XR_111050 when overexpressed enhanced osteogenesis of bone marrow MSC (Zhang et al., 2017).

Another report also demonstrated, after 14 induction days, a great number of regulated lncRNAs of which 88 showed an altered expression of more than 10-fold (55 up and 33 downregulated) (Huang et al., 2017). The most modulated lncRNAs were *uc002lbc.1* and *uc.247+*, up and downregulated, respectively. The same study also analyzed the mRNA profile, finding a great number of downregulated genes (Table 1). Despite that, GO analysis of upregulated genes was in agreement with other studies presenting, e.g., ECM organization as one of the main biological processes (Huang et al., 2017). The network of mRNA-lncRNAs highlighted 12 lncRNA interacting with more than 150 mRNAs, as FOXO1, GPM6B, FGF6, OMD, WNT5B. Specific study of lncRNA H19 showed that its expression was reduced during osteogenesis and that its knockdown resulted in the increased expression of osteogenic markers (Huang et al., 2017).

This dataset, generated by Huang et al. (2017), was reanalyzed and new observations regarding the lncRNA-mRNA-miRNA interaction were made. Many genes modulated during osteogenesis were related to the PI3K/Akt signaling pathway, among which IL6 was one of the overrepresented. Co-expression analysis showed a possible interaction with the lncRNA HIF1A-AS2, which in turn could be interacting with miRNAs, including miR-665 (Wu et al., 2018). Silencing and overexpression experiments of these molecules indicated a relationship between HIF1A-AS2 - miR-665 - IL6 and that this core regulates the PI3K/Akt signaling pathway (Wu et al., 2018). In addition, another pathway explored was the toll-like receptor (TLR) signaling pathway. Using similar strategies to previous study (Wu et al., 2018), Yu et al. (2018) observed an interaction between TLR4 (upregulated in osteogenesis) with lncRNA-PCAT1 and miR-145-5p, which were able to regulate the TLR pathway.

Although the function of circRNAs is not completely understood, it is known that they could act in the regulation of gene expression – including pathways related to osteogenesis (reviewed by Huang X. et al., 2019) -, regulating, for example, the expression of its host gene or even function as a miRNA sponge (reviewed by Santer et al., 2019; Yu and Kuo, 2019). Thus, combined analysis of mRNA, miRNA and circRNA have been carried out in an attempt to set up interaction networks and understand how they can contribute to the regulation of osteogenic differentiation.

The number of differentially expressed circRNAs was variable between studies: while one identified over 2000 differentially regulated circRNAs after 7 days (Zhang M. et al., 2019), 2019), other found ∼100–150 circRNAs modulated at different time points of osteogenic differentiation (3, 7 and 14 days) (Zheng et al., 2017). Despite this, the GO analysis both of the mRNAs and of the parental genes of differentially expressed circRNAs highlighted terms related to osteogenesis such as ECM, cell differentiation, plasma membrane, cytoplasmic or membrane bound vesicles and others (Zheng et al., 2017; Zhang M. et al., 2019). Through the construction of a miRNA-circRNA interaction network, the possible relationship between circIGFS11 and miR-199B-5p was indicated: while one has a reduction after 7 days of differentiation, the other increases its expression. Functional tests confirmed that silencing circIGSF11 increased the expression of miR-199b-5p and was able to induce osteoblast differentiation (Zhang M. et al., 2019). The construction of interaction networks also made it possible to identify circRNAs that interacted with miRNAs previously described as having a role in osteogenesis (Zheng et al., 2017). The description of these circRNAs already indicates that they are modulated throughout differentiation, potentially involved in the regulation of their host genes and miRNAs that have positive effects in processes related to osteogenic differentiation.

Thus, it is possible to note the complexity of the osteogenesis process and how the use of transcriptome studies helps to understand it. The identification of biological process or signaling pathways regulated over the differentiation process indicated those that are critical to the osteogenic process, such as those related to the ECM-organization, MAPK and PI3K/Akt pathways. In addition, miRNAs, lncRNAs and circRNAs are an emerging source for the comprehension of regulatory mechanisms of osteogenesis. All these works contribute to the development of the field, as well as helping to understand diseases related to, for example, bone development.

## Gene Expression Profile in Chondrogenesis of MSC

The hyaline cartilage is responsible for the bone formation in the embryo (thought endochondral ossification), and in adults can be found in costal cartilages, respiratory system, and covering the bone articular surface (reviewed by Carballo et al., 2017). The treatment for cartilage defects, e.g., articular hyaline cartilage defects, is a challenge and the MSC appeared as an alternative for cartilage engineering, since it has the ability to differentiate into chondrocytes *in vitro*. The process of chondrogenic differentiation of MSC is commonly performed with pellet or aggregate culture system, with addition of factors as TGFβ, BMP and/or IGF (reviewed by Boeuf and Richter, 2010; Somoza et al., 2014; Table 1). Chondrocytes generated from MSC expressed classical genes/proteins as native chondrocytes, e.g., type II collagen and aggrecan. However, it is also possible to identify hypertrophy-associated genes, as type X collagen, ALP and MMPs (reviewed by Hellingman et al., 2012; Somoza et al., 2014). Thus, differently from normal hyaline cartilage, the *in vitro* differentiation process seems to arrest in early phases of endochondral ossification (Pelttari et al., 2006; Steck et al., 2009; Somoza et al., 2014). Although differentiation protocols are still unable to generate a type of cartilage that resembles articular cartilage in normal physiological conditions of an adult organism, understanding the stages of chondrocyte commitment and comparing it with fetal or adult cells can be of great help in improving the *in vitro* chondrogenesis of MSC.

Since the ECM elements are essential components of cartilage tissue, high expression of ECM related genes, mainly collagens types, was detected in many studies (Ikeda et al., 2007; Djouad et al., 2009; Mrugala et al., 2009; Somoza et al., 2018; Huynh et al., 2019). For example, Ikeda et al. (2007) used the microarray technology to determine the gene expression profiles of MSC following monolayer chondrogenesis after 14 days of induction. The authors identified 23 upregulated and 35 downregulated transcripts, of which 44 and 40%, respectively, were associated to ECM and metabolic pathways. Many collagen types were identified as up (COL10A1, COL11A1) or downregulated (COL6A3), as well as other components uprelated to ECM suchas CLU, SAA1, PTX3, MGP (Ikeda et al., 2007). Interestingly, pathway related to cell growth presented more downregulated genes, e.g., IGFBP2, PDGFB, EMP1, PDGFRA (Ikeda et al., 2007). The last one downregulated only after 3 days of chondrogenesis (Somoza et al., 2018).

In an attempt to identify new factors responsible for chondrogenic differentiation, a gene expression profile of MSC following BMP2-induced chondrogenesis (micropellet) over a 21-day period were performed (Djouad et al., 2009). As expected, the mRNA expression levels of many collagens types characteristics of cartilage tissue (as COL2A1, COL9A2, COL9A3, and COL11A1) were increased in late stage chondrogenesis. Also, aggrecan and cartilage oligomeric protein were upregulated, which are highly and specifically expressed in cartilage, validating the differentiation process. Furthermore, despite the identification of previously known transcription factors associated with chondrogenesis, as SOX9, Twist1 and TCF1, five novel transcription factors were upregulated in differentiation process: FOXO3A, Dlx4, Nessy, Sox13, and Tbox6. Among them, FOXOA3 was shown indeed to be involved to differentiation and apoptosis during chondrogenic differentiation of MSC (Djouad et al., 2009).

Considering the importance of initial signaling in differentiation processes, Gong et al. (2018) performed an analysis of MSC after only 3 days in chondrogenic induction media. Among the genes with a markedly increased expression were DYNC1I1, BNC2, ENPP1, FBXO42, JMYN, FATC1, and PLCE1, while the expression of DNMT3A, PLCG2, ANXA11, GRK6, HSP90B1, KEAP1, and NDST2 were downregulated (Gong et al., 2018). In the GO and pathway analysis, T cell receptor signaling and antigen receptor-mediated signaling were overrepresented while the underrepresented processes included skeletal system, osteoclast differentiation and acute inflammatory response (Gong et al., 2018).

As an effort to investigate the specific molecular signature during chondrogenesis of MSC and elucidate the dynamic of differentiation process, Mrugala et al. (2009) performed a microarray analysis after 1, 3, 7 and 21 days of chondrogenic differentiation, using TGF-β3 or BMP2 induction medium. In addition, adipogenesis and osteogenesis of MSC were also performed and analyzed at the same time points (Mrugala et al., 2009). Comparing the data and selecting the genes that appear only in cells with chondrogenic induction, 318 genes were found as differentially expressed, of which 177 were known sequences. Based on gene expression profile, it was characterized the phases of chondral differentiation: 1) cell attachment and apoptosis, represented by genes as BCL6, ITGA5, NFIL3, CTGF; 2) differentiation induction, including genes such as Wnt5a, Notch3, FOXO1A, FOXO3A, IGFBP1; and 3) Wnt signaling inhibition and hypertrophy with upregulated genes like FKBP5, SLUG, TIMP4, DKK1, APOE/D (Mrugala et al., 2009). Another analysis demonstrated that angiopoietin-like 4 (ANGPTL4) is upregulated during chondrogenesis, mainly at days 1 and 3. Addition of exogenous ANGPTL4 in TGF-β3-induced MSC decrease the expression of classical chondrogenic markers, as aggrecan, COL2A1, COL10A1, and increased the presence of MMPs. On the other hand, knockdown of this gene improved the micromass size and the expression of chondrogenic markers. These results indicated that ANGPTL4 regulates ECM components in chondrogenic differentiation (Mathieu et al., 2014).

Comparison of transcriptome data from *in vitro* MSC chondrogenesis and the normal articular cartilage could allow the identification of common or different signals and regulatory elements, indicating novel strategies that could improve *in vitro* chondrogenic differentiation. A high-throughput analysis of differentially expressed genes of MSC after 3, 7, 10, 14, 21 and 28 days of chondrogenic induction and the characterization of transcriptional regulatory elements from human neonatal articular cartilage showed that more than 500 genes that were highly expressed in neonatal cartilage were not expressed at any time point during *in vitro* chondrogenesis (Somoza et al., 2018). But, interestingly, it was observed that cells at early stages of differentiation (days 3 and 7) were more similar to neonatal cartilage than those from later days, suggesting that, at this time points, it is still possible to interfere and redirect the cells to a specific cartilage phenotype. The data analysis also demonstrated that MSC during chondrogenesis expresses classical markers of hyaline cartilage as aggrecan, SOX9, COL2 and others, but also expressed COL10, Runx2, ALPL, and MMP13 which are presented in hypertrophic cartilage. This indicates that the markers actually used could not be able to really distinguish an articular cartilage from those generated by MSC differentiation process (Somoza et al., 2018). Comparative analysis demonstrated that among the control elements identified as upregulated in neonate cartilage were the UCMA (Unique Cartilage Matrix-Associated Protein), MSMP (Microseminoprotein, prostate associated), MATN1 (matrilin 1) among others. Furthermore, pathways analysis of the differentially expressed genes in neonatal cartilage (182 up and 191 downregulated) indicated an enrichment in integrin related pathways, as well as those related to VEGFR, ErbB1, IGF1 and others (Somoza et al., 2018). Then these results confirmed the differences between a human cartilage and the *in vitro* chondrogenic differentiation of MSC, but highlight possibilities to improve the protocol.

Similarly, a temporal analysis of MSC after 1, 3, 7, 14, and 21 of chondrogenesis induction confirmed that the *in vitro* derived cartilage, at least in transcriptional level, was different from human articular cartilage from embryonic, adolescent or adult origin (Huynh et al., 2019). Besides, the greatest change in gene expression was observed between day 0 and day 1, with more than 2000 upregulated and 1860 downregulated genes. As expected, during differentiation induction, chondrogenic markers were upregulated as well as naïve MSC markers decrease its expression (Huynh et al., 2019). Gene co-expression network analysis identified a functional module composed of 1172 genes upregulated during differentiation. Analysis of this module indicated that the most enriched pathway was skeletal system development, nevertheless other pathways were also present as ECM and collagen fibril organization, demonstrating the chondrogenic profile. In addition, it was identified a set of transcription factors, including members of SOX family, retinoic acid receptor, FOS/JUN complex and FOXA2, and 230 lncRNAs (Huynh et al., 2019). As previously related (Ikeda et al., 2007), cell proliferation was upregulated at initial time points and downregulated at late stages, while pathways related to chondrogenesis, especially those related to ECM, were upregulated during the entire process at all time points analyzed (Huynh et al., 2019).

Recently several circRNAs, miRNAs and piRNA were differentially expressed after 7 days of chondrogenic and osteogenic induction (Della Bella et al., 2020). This analysis identified 130 up and 97 downregulated circRNAs in chondrogenesis, of which 15 were also identified in osteogenesis. Notably, many of the circRNAs identified share the same precursor gene, as FKBP5, FADS2, ZEB1, and SMYD3, which were also found in osteogenic induced cells. Investigating if the expression of these genes were influenced by dexamethasone, a component of both induction media (Table 1), the cells were exposed only to the compound in monolayer or pellet culture. The results indicated that while the expression of FKBP5 was affected by the presence of dexamethasone, FASD2 gene showed no alteration in its levels (Della Bella et al., 2020). FASD2 and FKBP5 were previously identified in chondrogenic induction. The first showed increased expression after 14 days of induction (Ikeda et al., 2007), while FKBP5 was upregulated during chondrogenesis process (Ikeda et al., 2007; Mrugala et al., 2009). Interestingly, the composition of induction medium is different between these studies, not all containing dexamethasone (Table 1).

Regarding miRNAs, more than 200 were identified as differentially expressed (102 up and 108 downregulated) in chondrogenic differentiation (Della Bella et al., 2020). Its mRNA targets had relation to PI3K-AKT signaling pathway, NK-kappa B signaling pathway and others. Furthermore, the identification of miRNAs allowed to relate them with the circRNA expressed in chondrogenesis, once they can act as miRNAs sponges. For example, some of circRNAs with binding sites for hsa-miR-665 were upregulated (hsa_circRNA_081069, hsa_circRNA_100833, hsa_circRNA_002161), while the miRNA was downregulated (Della Bella et al., 2020). An interesting observation is that while in osteogenesis only 54 piRNAs were differentially expressed, in chondrogenesis it was identified 131 piRNAs, the most part upregulated (73). But future studies need to be performed to understand the role of this class of RNA in differentiation process (Della Bella et al., 2020).

The data discussed here indicated that, despite the advances, the protocols developed markedly generated chondrocytes with hypertrophic phenotype, generating a cartilage-like tissue different from normal articular cartilage. However, using this information could help to identified the key points that needed to be improved and also new markers that characterize the chondrocytes generated *in vitro* from MSC.

## One by One: Single Cell Transcriptomics of Human MSC

Transcriptomic analysis is usually performed in tissue samples, populations of isolated cells or in cells in culture. In every case, there is the assumption that the samples are homogeneous and each cell responds in a similar way to a given stimulus. However, we now know that every cell in a population has a particular response depending on their cell cycle, metabolic state and environmental or positional information. This is of particular concern when studying MSC gene expression profiles. Human MSC have been defined as a heterogeneous population with subpopulations differing in their multipotency and, hence, being a challenge for transcriptome characterization (Wagner et al., 2006; Russell et al., 2010). In this context, single-cell analysis could be a way to bypass the worries of studying heterogenous cell populations.

The combination of FACS or microfluidic cell isolation and high-throughput sequencing, allows the identification of gene expression profiles of isolated cells from a target population (Li et al., 2013; Hedlund and Deng, 2018). First approaches combined single cell isolation and RNA extraction, with expression analysis of a defined set of genes by qPCR or by interrogating microarray devices (e.g., Acosta et al., 2017; Hardy et al., 2017; Khong et al., 2019; in MSC). However, the emergence of next generation sequencing methods enabled transcriptomic studies of single cells to reach higher levels of coverage. Tang et al. (2009) reported the first single cell transcriptome assay describing the gene expression patterns of cells from murine oocytes and blastomere (Tang et al., 2009). Since then, single cell RNA-seq (scRNAseq) has been applied to analyze a wide range of cell populations under different biological conditions (Hedlund and Deng, 2018; Hwang et al., 2018).

scRNAseq assays in MSC focused mainly on the characterization of the heterogeneity of the isolated populations and in defining the gene expression patterns of cells from different sources. Liu et al. (2019) performed a large scale RNAseq of 24,370 adipose tissue-derived MSC from three different donors. Interestingly, they observed that most of the heterogeneity observed was due to batch effect and cell cycle phase of the cells. After removing the batch and cell cycle effect they obtained a clean gene-expression matrix that is available for further characterization (Liu et al., 2019). In another work, adipose tissue-derived MSC were compared to bone marrow-derived MSC from the same donor. Adipose tissue-derived MSC showed lower transcriptomic heterogeneity, though different subpopulations were observed. Moreover, adipose tissue-derived MSC were less immunogenic with higher immunosuppression capacity (Zhou et al., 2019). Also, in adipose tissue-derived MSC isolated from perivascular adipose tissue two defined subpopulations could be identified after scRNAseq one of them with higher potential to differentiate into smooth muscle lineages (Gu et al., 2019). On the other hand, umbilical blood MSC (hUC-MSC) showed limited heterogeneity even after stimulation with different cytokines. As mentioned before, most of the heterogeneity observed was related to the cell cycle stage of the cells (Huang Y. et al., 2019). Jia et al. (2020) reported opposite results, identifying several clusters in hUC-MSC (Jia et al., 2020). These discrepancy in the results could be due to differences in cell isolation methods or bioinformatic analysis.

Two reports studied gene expression in human primary Wharton’s jelly-derived MSC (hWJMSC) by scRNAseq revealed the existence of several distinct subpopulations of MSC. These subpopulations exhibited diverse functional features related to proliferation, development, and inflammation response (Barrett et al., 2019; Sun et al., 2020). Batch effects and cell cycle stage of the cells must be considered, as they can result in major changes in the gene expression patterns observed (Liu et al., 2019; Sun et al., 2020). Even though, scRNAseq emerge as a powerful tool to address the differentiation potential of the subpopulations found among MSC and could also be used to investigate different cell differentiation stages in the differentiation processes.

## Perspectives

The use of stem cells in therapies has gained interest over the past few years. One of its characteristics is the potential for differentiation into mesodermal lineages that include adipocytes, chondrocytes and osteoblasts. The mechanisms of cell differentiation are complex and, despite the advances in the knowledge of the processes, the mechanisms regulating them are not yet fully understood. High-throughput analysis, e.g., transcriptome and translatome, are strategies helping to shed light on the molecular events driving osteogenesis, chondrogenesis and adipogenesis.

Although the advances shown in this review, challenges remain. The variety of differentiation protocols, cell origin, the investigated time points, RNA type used for analysis and sequencing methodology generates a large amount of data that exhibit significant variations in results. On the other hand, these differences can be important to determine which are the determining factors, regardless of the condition, that could stimulate cell differentiation. The studies covered in this review, and still others that have not been cited, contribute to the understanding of the key events, molecules and pathways that lead to adipogenesis, chondrogenesis and/or osteogenesis, as well as in the comprehension of related diseases and the indication of possible therapeutic strategies.

## Author Contributions

All authors listed have made a substantial, direct and intellectual contribution to the work, and approved it for publication.

## Conflict of Interest

The authors declare that the research was conducted in the absence of any commercial or financial relationships that could be construed as a potential conflict of interest. The handling editor declared a past collaboration with one of the authors BD.
